# epiTOol: a browser-based HLA epitope (mis)matching application for small to large cohorts

**DOI:** 10.3389/fimmu.2026.1916548

**Published:** 2026-09-07

**Authors:** Claudia Lehmann, Nils Lachmann, Ilias Doxiadis, Henry Loeffler-Wirth

**Affiliations:** 1 Institute for Transfusion Medicine, HLA Laboratory, University Hospital Hamburg-Eppendorf, Hamburg, Germany; 2 Institute for Clinical Immunology, University of Leipzig, Leipzig, Germany; 3 Institute for Transfusion Medicine, H & I Laboratory, Charité - Universitätsmedizin Berlin, Corporate Member of Freie Universität Berlin and Humboldt Universität zu Berlin, Berlin, Germany; 4 Interdisciplinary Centre for Bioinformatics (IZBI), Leipzig University, Leipzig, Germany; 5 Bioinformatics Group, Department of Computer Science, University of, Leipzig, Leipzig, Germany; 6 Medical Department III - Endocrinology, Nephrology, Rheumatology, University of Leipzig Medical Center, Leipzig, Germany

**Keywords:** epiTOol, eplets, HLA-antibodies, interactive epitope matching, predicted and confirmed epitopes

## Abstract

**Method:**

epiTOol is a web-based application for transplantation immunogenetics and immunology, providing easy and reliable translation of high-resolution HLA typing into either predicted or confirmed epitopes. Unacceptable or acceptable HLA incompatibilities or mismatches are then easily definable, and the data is exported for further analyses. Unlike other similar tools, epiTOol allows for upload and analysis of large datasets of patient-donor combinations without the need for manual data entry. The results can be directly exported in table and text format for subsequent utilization in medical reports or data sheets.

**Conclusion:**

epiTOol is designed for utilization in research activities dedicated to transplantation immunology in respective centers dealing with solid organ transplantation but will also be applicable to stem cell transplantation since the role of HLA-specific antibodies also increases dramatically here.

## 1 Introduction

The presence of HLA-specific antibodies targeting the organ donor in solid organ transplantation endangers long-term graft survival. Assessing acceptable and unacceptable HLA mismatches, together with the associated epitopes, is complex and necessitates expertise, proficiency, and especially accurate HLA typing. Previously, we demonstrated the advantages of high-resolution definition of the HLA alleles for an adequate identification of these antibodies and emphasized that concentrating on epitopes can facilitate optimal antibody definition and organ allocation (1–6).

However, the utilization of integrated, interactive applications for processing HLA data and generated antibody screening data is necessary: In addition to the epitope database (7, 8), a significant resource of previously defined or predicted HLA-allele determining amino acids (eplets), various analytical methods were introduced (7, 9–14). They present a broad set of options, nonetheless, in our view, they lack interactivity, particularly in the context of single-pair and bulk-analysis configurations. Current tools do not meet requirements of laboratories engaged in transplantation, which need simple, failure-robust, and reliable handling of the generated data when several donors are accessible for the patient.

Here we introduce ‘epiTOol,’ an interactive toolbox for comprehensive analysis of HLA typing and antibody data. We demonstrate functionalities and application of the program on a retrospective data collection of 143 living kidney transplants from the University of Leipzig Medical Center (Germany). In this example cohort, both patients and donors were HLA typed at high resolution for 11 HLA loci (15).

## 2 Materials and methods

### 2.1 Cohort description

Application of epiTOol is demonstrated using a living kidney transplant cohort comprising 143 pairs, as described in detail in Lehmann et al. (1). The donor/recipient pairs underwent a living donor kidney transplantation at the University of Leipzig Medical Center (Germany) between January 1998 and December 2018. In short: 59.9% of patients who received a living-donor kidney transplant were male, and approximately 58.7% of donors were relatives. *De novo* donor specific-antibodies (DSA) were detected in 43.1% of patients during follow-up screening.

### 2.2 HLA-typing and antibody profiling

The patient/donor pairs were typed for 11 HLA loci using an NGS protocol with Illumina short-read technology (SBS, sequencing by synthesis, San Diego, CA, USA), in accordance with the manufacturer’s instructions. The library was prepared using the QIAseq FX kit (Qiagen, Venlo, The Netherlands) and analysed using NGSengine software (GenDx, Utrecht, The Netherlands). The detailed HLA typing protocol can be found in Lehmann et al. (1).

HLA antibody assessment was conducted on a Luminex^®^200 platform utilizing the LABScreen™ Single antigen assays supplied by One Lambda, West Hills, CA, USA, in accordance with the manufacturer’s guidelines. A mean fluorescence intensity (MFI) of **≥** 1,500 was considered positive. All data were analysed utilizing the Fusion software v4.4 (1).

### 2.3 epiTOol import & export functionalities

The epiTOol prototype is implemented as an interactive web application using the R-Shiny framework. Future development towards a standalone software or an add-on to Laboratory Information Management Systems (LIMS) is under consideration. For data import, the tool accepts spreadsheet (Excel) files, which were exported from a database, LIMS, or elsewhere. These files contain HLA typing of a set of patients without limitation to single individuals or a maximum number. Future direct data transfer or embedding of epiTOol into LIMS is subject to development. Import of antibody profiling data is realized in terms of uploading the MFI values for the single-antibody assays for HLA class 1 and 2 from the Fusion software. Requirements of the data table formats are simple: the first column is required to contain any kind of identifier for the patients, either names, numbers, or other pseudonyms. Allele columns are named A#1, A#2, etc; antibody MFI columns A*01:01, A*02:01, etc. Functionalities will be extended to other manufacturers and also non-classical alleles (e.g. MICA, HLA-E, G & F) in future versions. Convenient export functionalities of the tables of epitope information and of match and mismatch analyses comprise options for spreadsheet (Excel) and PDF files, as well as a copy-paste function to include results into any documents, for example word files and report templates.

### 2.4 Epitope-based matching & mismatching

HLA-allele information is translated into epitopes using HLA Eplet Registry (7), which collects configurations of polymorphic amino acids on the HLA molecular surface. These ‘eplets’ are, in our opinion, the best approximation to determine an ‘epitope’, a specific antigenic determinant of an antigen that is recognized and bound by an antibody (16).

epiTOol offers calculation of mismatches between potential donor-recipient-pairs on epitope level, analogous to classical mismatch determination on HLA-allele level. For this, only antibody-confirmed eplets or all predicted eplets in the HLA Eplet Registry database can be utilized as options. Additional to mismatch analyses, epiTOol offers also epitope-based matching, i.e., extracting and providing epitopes shared between two individuals.

Epitope-based matching & mismatching can be applied in two different modes: i) One-versus-list: Here, a newly available donor can be compared to a whole list of potential recipients. Another application is the comparison of one patient to a list of potential donors, such as close relatives in case of living kidney transplantation. ii) Pairwise: This mode is suited for retrospective evaluation of donor-recipient-pairs especially in reports and research context.

## 3 Results

### 3.1 Import functionalities

**Figure 1:**
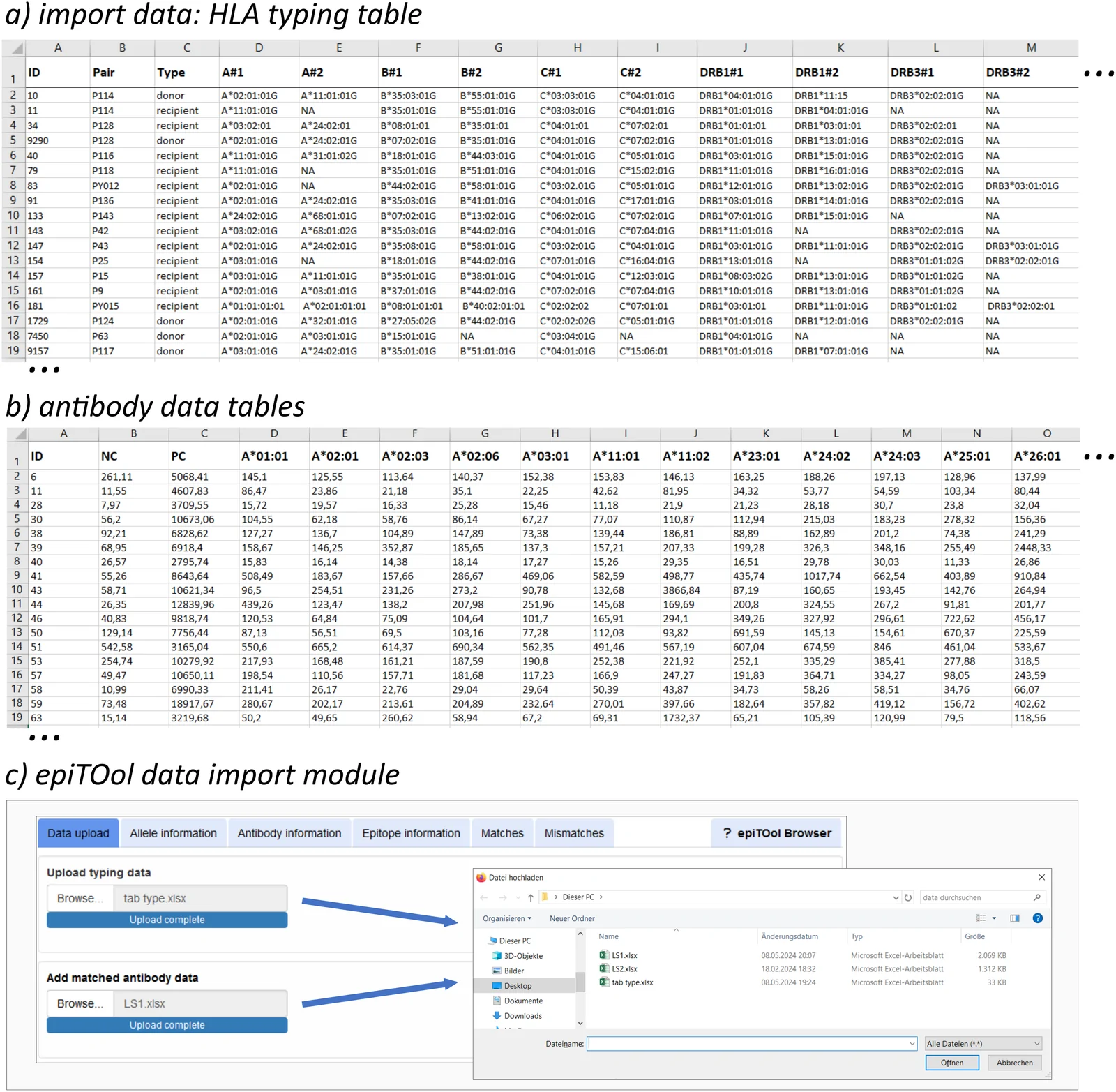
Data import into epiTOol: **(a)** Example of an HLA-typing spreadsheet. The first column is required to contain an alphanumeric ID, name, or any other pseudonym to identify each individual in the data. The second and third columns, as shown here, may contain information about donor-recipient-pairs necessary for paired epitope (mis)matching mode. The following HLA-allele columns are automatically detected by their ‘#1’ and ‘#2’ suffixes. Additional data, such as measurement dates, tray IDs etc. may be contained in the table and do not disturb data import. **(b)** Example of Luminex LS1 antibody data: The first column contains the identifiers that are used to link antibody profiles to the typing data. Bead MFI columns follow the name scheme ‘locus*group:allele’ and are automatically detected by the tool. Additional data, such as NC (negative control) and PC (positive control) beads may be contained in the table. HLA-class II antibody data tables are supposed to follow the same design. **(c)** Screenshot of the data import layer of epiTOol: HLA-typing and antibody data spreadsheets can be located on the local file system and subsequently uploaded into epiTOol.

To perform analyses in epiTOol, high-resolution HLA typing data for patient-donor pairs, as well as anti-HLA antibody data, must first be imported. Many other tools require manual insertion of data into forms, which is usually performed via copy-paste from an external spreadsheet. This procedure is, however, prone to errors and not suited for larger lists of individuals. Driven by our experience in routine diagnostics and subsequent data handling, we opted for a data import interface directly able to read and process Excel spreadsheets in the way they are generated by our Laboratory Information Management Systems (LIMS). An example of an HLA typing spreadsheet is shown in **Figure 1a**: In this format, the first column contains an alphanumeric ID, here a number, to identify each individual in the data. The further columns may contain information about donor-recipient pairs necessary for paired epitope (mis)matching modes. There is no specification in which columns this information is to be stored, and also additional data (measurement dates, tray IDs, etc.) may be contained in the table without disturbing the data import. HLA-allele columns are automatically detected by their ‘#1’ and ‘#2’ suffixes.

Luminex antibody data can also be imported from Excel files. **Figure 1b** shows an example table: Again, the first column needs to contain the identifiers, which are then used to link antibody profiles to the typing data. Bead MFI columns follow the name scheme ‘locus*group:allele’ and are automatically detected by the tool. HLA-class I and II antibody data tables follow the same design and can be uploaded separately or in a joint table. In general, epiTOol is not restricted to particular manufacturers, as long as the submitted data files follow the specified format. Example data files can be found on the tool’s webpage: http://epitool.bioinf.uni-leipzig.de/.

**Figure 1c** shows a screenshot of the epiTOol data import interface: Here, the user finds two upload buttons, one for HLA typing and one for antibody data. If multiple files are uploaded consecutively, the data will be appended. Upload of HLA typing is necessary to perform epitope matching and donor-recipient comparisons. Uploading antibody data, in turn, is optional and unlocks antibody information complementary to epitope comparisons.

### 3.2 Information functionalities

**Figure 2:**
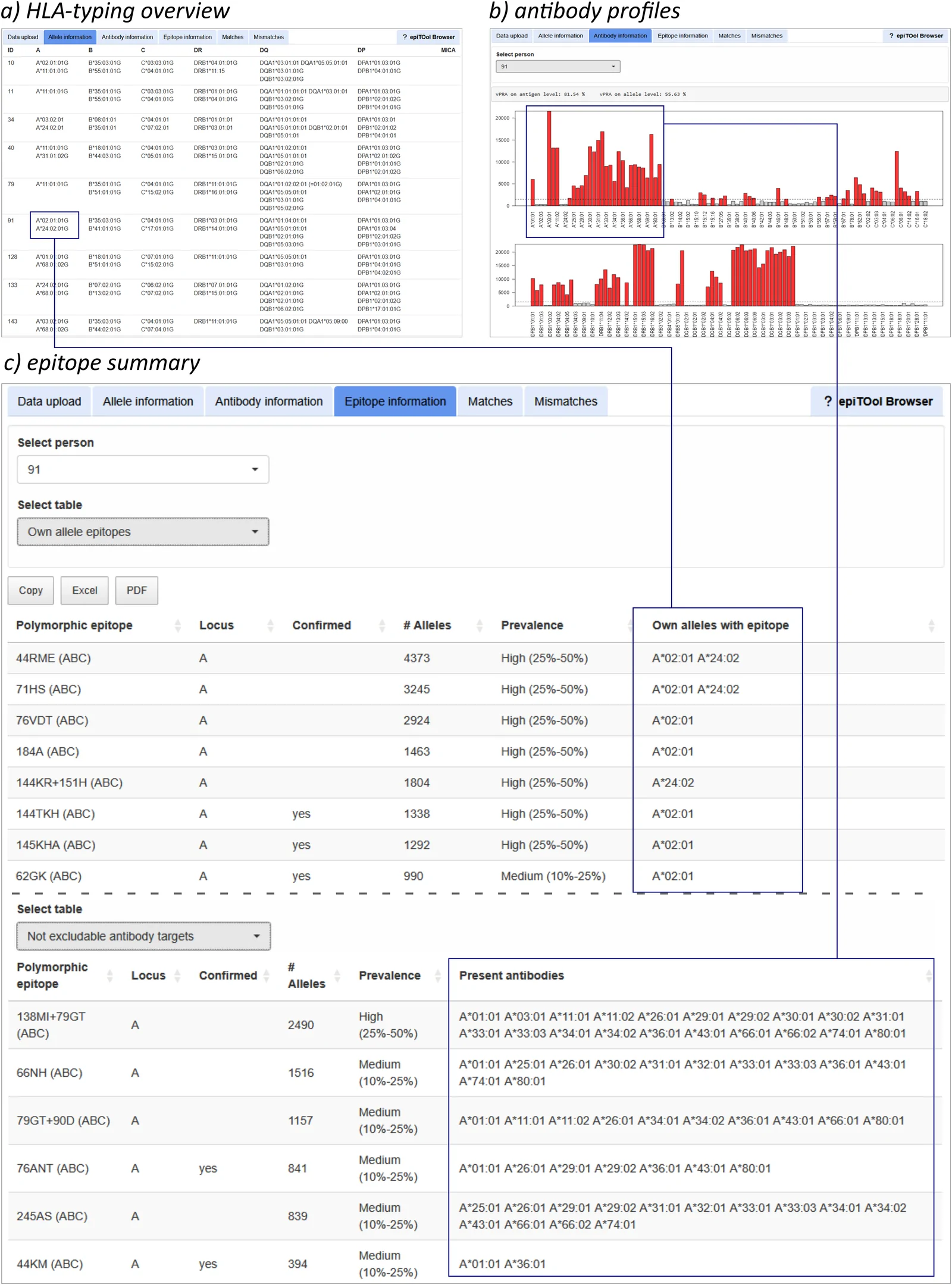
Information layer of epiTOol: **(a)** Overview of the HLA-alleles of each individual extracted from the typing table. This functionality serves as safety check to validate correct data import. **(b)** Barplots of HLA-class I and II antibody profiles of a selected individual are shown, if data is provided. Antibody beads exceeding a user-defined MFI value (standard: 1,500) are highlighted in red colour. Additionally, vPRA-values are given. **(c)** Epitope summary of a selected individual: Epitopes reported for the persons HLA-alleles are listed together with information on locus, confirmation status, number of alleles associated with this epitope, and the prevalence of the epitope in a German reference cohort of ≈35,000 individuals (top part) (17). For antibodies, epitopes related to beads with MFI >1,500 cannot be excluded as ‘present in the serum’ and are listed together with the additional epitope information (bottom part).

After HLA typing data is imported, several overview options of the data can be displayed. The first step is to check that HLA-typing is processed correctly by inspecting the overview of the HLA-alleles of each individual extracted from the typing table (**Figure 2a**). Then, in case that antibody data was uploaded to the tool, antibody information overviews can be generated presenting barplots of HLA-class I and II antibody profiles of a selected individual (**Figure 2b**). Antibody beads exceeding MFI of 1,500 are considered as positive and highlighted in red colour. This threshold can be modified according to the user’s protocols and guidelines. The positive antibody beads will also be used in the epitope summaries and (mis)matching results (see below).

For each individual, epitopes reported for the corresponding HLA-alleles are listed in an epitope summary overview (**Figure 2c**, top part). This table also provides information on locus of the epitopes, confirmation status, number of corresponding HLA-alleles, and the prevalence in a German reference cohort of ≈35,000 individuals (17). A second option is to show antibody epitopes for the person’s positive antibody beads (**Figure 2c**, bottom part). Usually in single-antigen bead approaches, it is not clear to which of the multiple epitopes on an HLA molecule present antibody eventually bound. In consequence, beads with high MFI-values (here: >1,500) indicate presence of antibodies against at least one of the epitopes on the molecule, such that all epitopes of the bead cannot be excluded as ‘present in the serum’. epiTOol presents epitopes derived from antibody profiling in this way, and they are listed together with the additional information in the epitope overviews (**Figure 2c**, bottom part).

### 3.3 Eplet information – confirmed and not confirmed

Eplets and epitopes are related but not identical concepts. An ‘epitope’ is the antigenic site recognized by an antibody, usually understood as the structural region on an antigen that participates in binding. An ‘eplet’ is a smaller molecular configuration, typically a limited set of polymorphic amino acid residues that represents the essential structural component of an HLA epitope and contributes to antibody recognition. Methodically, we approach information on epitope level by using eplets as a proxy. These eplets are collected and thoroughly characterized in the HLA Eplet Registry database (7), which is used by epiTOol to assign epitopes to the HLA-alleles and antibodies in the data.

The distinction between ‘confirmed’ and ‘not yet confirmed’ epitopes is useful because it separates experimentally validated antibody targets from structures that are only predicted or inferred in silico from the DNA sequence and modelling. This improves scientific rigor, helps avoid over interpretation of theoretical assignments, and gives clinicians and researchers a more reliable basis for comparing HLA mismatches, risk stratification, and antibody cross-reactivity. We support both, a more stringent or a more explorative epitope screening: In epiTOol, it is consequently possible to switch between antibody confirmed and all (including theoretical) eplets.

### 3.4 Epitope (mis)-matching functionalities

**Figure 3:**
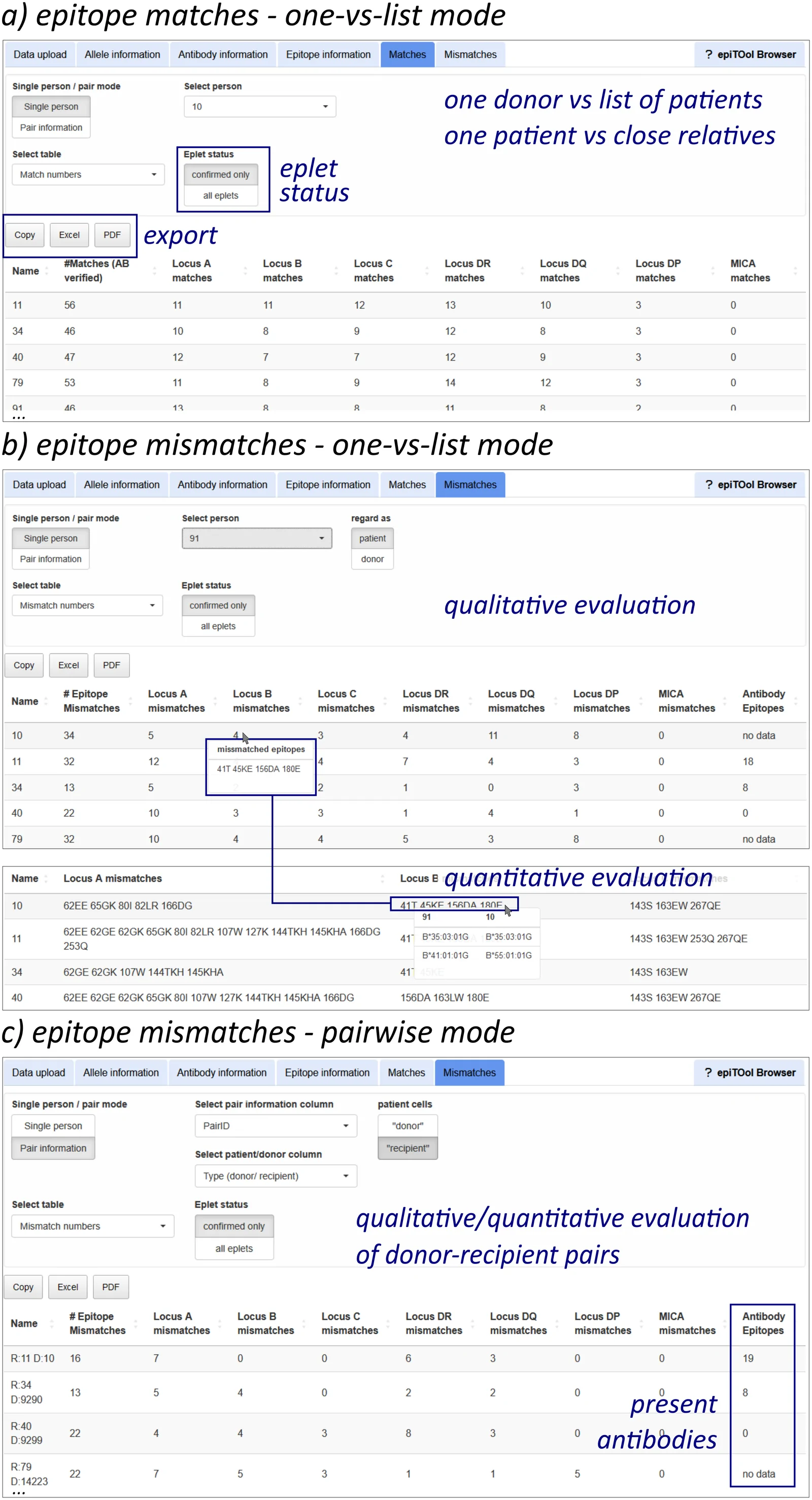
Epitope (mis-) matching functionalities of epiTOol: **(a)** Epitope match numbers between potential donors and recipients are listed by locus and can be exported to clipboard, excel or pdf file. Here, the one-versus-list-mode is shown, comparing a selected individual against all other persons in the data imported into epiTOol. **(b)** Epitope mismatches can be listed analogously, whereby it is required to provide information if the selected person is a patient or a potential donor. Both match and mis-match analyses provide qualitative and quantitative information, i.e., epitope numbers as an overview and locus-wise lists of the epitopes, respectively (see top and bottom part). **(c)** Epitope mismatches in pairwise mode: Additional information about donor-recipient-pairs were provided in the typing table and allow direct comparison of each of these pairs here. This mode is also available for epitope matches.

epiTOol allows for donor-recipient comparisons in terms of matched (common) epitopes and epitope mismatches (differences) in analogy to the HLA-allele mismatches established in transplantation immunology (**Figure 3**). Furthermore, epiTOol provides two different modes to compare donors and recipients: In the ‘one-versus-list’-mode, one person can be compared to a list of other persons. For example, a newly available organ can be screened against a waiting list of patients, or a kidney patient can be compared to multiple potential living donors such as close relatives. The pairwise mode, on the other hand, uses a pair-ID in the HLA-typing data and then compares donor and recipient for each pair instance. This mode is especially useful in research settings and enables, e.g., retrospective evaluation of large cohorts of transplanted donor-recipient-pairs.

**Figure 3a** shows an example epitope matching analysis, comparing the selected person with all other persons in the data uploaded. The resulting epitope match numbers are listed by locus and can be exported to clipboard, excel or pdf files. **Figure 3b** shows a similar epitope mismatch analysis. Both example screenshots show the quantitative view on the respective comparison, i.e., match and mismatch numbers. They are supplemented by locus-wise tables of (mis)matched epitopes as their qualitative counterparts (**Figure 3b**, lower part). A screenshot of the pairwise mode is exemplarily shown in **Figure 3c**. If antibodies are present in the recipient, potentially conflicting epitopes (i.e. donor-specific epitopes - DSE) of these antibodies are additionally provided by epiTOol.

In summary, the user can compare epitopes between donor(s) and recipient(s) based on matches and mismatches, work in one-vs-list-mode or pairwise mode, and investigate qualitative and quantitative information as presented in epiTOol. All of these options can be applied using either all previously reported eplets in the HLA Eplet Registry database, or using only experimentally confirmed eplets.

## 4 Discussion

epiTOol is an interactive tool for all-in-one processing of HLA typing and antibody data in diagnostic workflows and scientific evaluation. First analyses of patient-donor pairs were presented in Lehmann et al. (15). All essential information about the tool, file templates, and a brief registration form is available on our website: http://epitool.bioinf.uni-leipzig.de/.

The tool is straightforward and allows both patient by patient specific and bulk analyses. It is therefore usable for the decision-making process in selection of a suitable donor in case of a living transplant, or a patient in case of a cadaveric donor. Furthermore, it allows bulk analyses in case of need for interpretation of the results, and comparison and inclusion of the data with e.g., transplantation data bases. Compared to the currently available algorithms (2, 4, 5, 7, 10, 12, 13) epiTOol is simple to use and allows import and export of data and analyses in standardized ways. The data can be then incorporated and used further adding e.g., transplantation data or disease data. The use of the HLA Eplet Registry (7) data base is essential since it provides the current base for eplets. However, in case the community decides to switch to another registry this is also possible. Being able to use between ‘confirmed’ and ‘not yet confirmed’epitopes in the matching and mismatching analyses allows the definition of possible new eplets influencing transplantation and antibody definition. To our opinion high resolution HLA typing is a prerequisite for correct eplet definition. epiTOol allows prospective and retrospective analyses of single pair or bulk data, offering upload of HLA typing and antibody results directly avoiding clerical errors. This type of errors was shown by Johnson et al. (12). The epiTOol can be used in other than kidney transplantation provided high resolution HLA typing and HLA antibody results. Therefore, it is suitable for virtual crossmatching in solid organ and/or stem cell transplantation. Furthermore, a patient and centre specific definition can be performed. Here, both listing of unacceptable for the patient HLA antigens can be defined, as well as delisting according to the local protocol (18), if needed. The unacceptable HLA antigens can be redefined as donor specific eplets (DSE).

Prospectively, we will extend the tool to support further pre-defined background cohorts for epitope prevalence and vPRA calculations, and to cover additional loci and alleles such as MICA and HLA-E, F and G.

The focus of this study is to demonstrate the functionality of epiTOol with a view to enabling a more detailed analysis of anti-HLA antibodies following transplantation. Limitations arise from the fact that the tool’s applicability has only been tested at a single centre on a cohort of living kidney donors. Its functionality and user-friendliness, particularly in a 24-hour setup involving post-mortem donors, should be revealed tested in a multi-centre setting in future.

Finally, epiTOol can be used in the case of broadly or highly sensitized patient for the identification and number of mismatched eplets irrespectively their position on the different class I or II loci. However, their immunogenicity cannot be attributed yet. epiTOol might be used as well in the living donation setup, e.g. kidney pair donation, for the prospective virtual crossmatch. In the future, the targeted prevention of HLA antibody formation may become feasible, provided that comprehensive data on the immunogenicity of individual eplets become available and findings are validated in larger, well-characterized cohorts. Since antibody screening is hampered by the increased number of new HLA alleles, the DNA sequence of those can be transformed into eplets in the same way as proposed earlier (9, 19). This might be the way to go.

## Data Availability

The raw data supporting the conclusions of this article will be made available by the authors, without undue reservation.
